# Functional Foods and Nutraceuticals in a Market of Bolivian Immigrants in Buenos Aires (Argentina)

**DOI:** 10.1155/2012/320193

**Published:** 2011-12-08

**Authors:** María Lelia Pochettino, Jeremías P. Puentes, Fernando Buet Costantino, Patricia M. Arenas, Emilio A. Ulibarri, Julio A. Hurrell

**Affiliations:** ^1^Laboratorio de Etnobotánica y Botánica Aplicada (LEBA), Facultad de Ciencias Naturales y Museo, Universidad Nacional de La Plata, Paseo del Bosque s/nro., La Plata, B1900 FWA, Buenos Aires, Argentina; ^2^CONICET, Ciudad de Buenos Aires, Argentina; ^3^Instituto de Botánica Darwinion (Academia Nacional de Ciencias Exactas, Físicas y Naturales-Consejo Nacional de Investigaciones Científicas y Técnicas, CONICET), Labardén 200, San Isidro, B 1642 HYD, Buenos Aires, Argentina

## Abstract

This paper presents the results of a research in urban ethnobotany, conducted in a market of Bolivian immigrants in the neighborhood of Liniers, Ciudad Autónoma de Buenos Aires (Argentina). Functional foods and nutraceuticals belonging to 50 species of 18 families, its products, and uses were recorded. Some products are exclusive from the Bolivian community; others are frequent within the community, but they are also available in the general commercial circuit; they are introduced into it, generally, through shops called *dietéticas* (“health-food stores”), where products associated with the maintenance of health are sold. On this basis, the traditional and nontraditional components of the urban botanical knowledge were evaluated as well as its dynamics in relation to the diffusion of the products. Both the framework and methodological design are innovative for the studies of the urban botanical knowledge and the traditional markets in metropolitan areas.

## 1. Introduction

This paper presents preliminary results obtained from a research line about Urban Ethnobotany developed in the Laboratorio de Etnobotánica y Botánica Aplicada (LEBA), Facultad de Ciencias Naturales y Museo, Universidad Nacional de La Plata. The object of study is the botanical knowledge (BK) in the main metropolitan area of Argentina, the contiguous urban agglomerations surrounding Buenos Aires, the capital city of the country, and La Plata, the capital city of the province of Buenos Aires. The composition and the dynamics of the BK are evaluated. This knowledge guides the selection and use of plants, their parts and products deriving from them found in the context of the conurbation.

This survey has been developed in a Bolivian immigrant market located in the city of Buenos Aires, which provides the specific products for this community and it is also representative of the pluricultural context of the metropolitan area [1, 2].

Researches on traditional markets have usually been addressed from the anthropology and the economic geography points of view as systems in which their components (actors and social networks, exchange and distribution, the products with their origin and destination) have to be explored [3]. It is important to note that in Latin America, markets represent valuable places for ethnobotanical researches as they condense in a reduced area the local knowledge and values on biological products. “Markets are public spaces devoted to sell several products, as well as they are spaces of exchange and acquisition of cultural information. Those spaces are walking traces of a determined culture or society by reproducing in a small scale, the biological and cultural diversity of a region” ([4], authors' translation). Consequently, they have been object of study, especially those in rural areas [5, 6] but there are only some previous investigations about markets placed in urban areas as the one described here [7]. It is considered a priority subject, because markets are true germplasm banks that help to preserve plant diversity through the use of different species [8]. The aim of this paper is to contribute to the ethnobotanical studies of markets in Argentina from the perspective of urban ethnobotany and to promote ideas for other aspects related to the value of the markets in connection with the understanding of the BK of the urban areas.

## 2. Materials and Methods

### 2.1. Framework

 Ethnobotany is a complex science due to both the diversity of issues and the variety of approaches that it includes. This plurality is framed in a broad concept of the discipline: the study of the relations between humans and their vegetal environment [9–11]. An aspect related to those relationships has acquired a central development: researches about *botanical knowledge* (BK), a set of knowledge and beliefs about the relationships between people and vegetal elements of their environment: plants, parts of plants, or products deriving from them.

Most of the surveys about BK have been oriented to societies called *traditional*. Even if this term is not exempt from discussion [12], it is considered that the *traditional botanical knowledge* (TBK), related to the concept of *traditional ecological knowledge* (TEK), is characteristic of culturally homogeneous contexts, with a long experience of human group in its environment; knowledge is transmitted from generation to generation orally and in the shared practices, and there is a direct link between production and consumption: those who consume produce [13–15]. Besides, the TBK is *adaptive*, because it allows adjustments of the group to the environmental changes; this is why it is not static or conservative but dynamic and innovative [16, 17]. There has been a rise in the number of studies about BTK, because they are usually endangered and their rescue is urgent.

On the other hand, the BK of the inhabitants of the urban agglomerations has been considered *nontraditional*; by contrast with the TBK, it corresponds to pluricultural contexts, with human groups without a large experience in the environment; knowledge is transmitted through social means of communication, and there is an indirect link between production and consumption: those who consume do not produce. The majority of the urban population knows little about the properties of the vegetal elements and less about their components or their origin, and the ways of obtaining and processing them are even less known [18]. However, this type of BK is also *adaptive*, because it guides the choice of what to consume [19]. Therefore, not long ago, several researches on urban ethnobotany came out based on studies about the BK of some part of the population of the conurbations. While some contributions on this field deal with plants product used by the average consumer in urban areas [1, 19, 20], up to now, most of the papers on urban ethnobotany are devoted to groups of immigrants that preserve a BK linked to their native traditions, which are readapted to their new context. In this way, there are several contributions from different parts of the world [21–33] and Argentina [2, 34–39]. 

The characterization of the BK of urban agglomerations is deficient if only the nontraditional BK is considered; together with it (including the scientific knowledge), different kinds of BK within the pluricultural context related to different traditions coexist: those of immigrants from various origin and those that belong to a part of the population that keeps their “family traditions”. The BK of these segments is linked to traditions, but it does not constitute a TBK in the sense defined above. Thus, what we call *urban botanical knowledge* (UBK) is a complex and adaptive corpus formed with a set of knowledge and beliefs about vegetal elements that coexist and interact within the same pluricultural scope [1, 2, 37]. 

On this basis, urban ethnobotany gives an answer of how is the *composition *of the UBK, that is, which are its components: linked to traditions and nontraditional and what is its *dynamics*: how the transmission of knowledge about the vegetal elements and their uses take place in the studied area. Several plants, their parts, and products deriving from them are *visible* for all the urban population and belong to the general commercial circuit, and their uses are widespread by the media; other plants remain restricted to immigrant groups or to the sphere of familiar tradition, and they are *invisible* for the majority. Nevertheless, some of these vegetal elements become visible when they enter the general circuit. In terms of the UBK dynamics, a component of the restricted BK (linked to traditions) spreads, and it gets generalized.

### 2.2. Functional Foods and Nutraceuticals

In this paper, the survey data focused on species that are used, at the same time, for food and therapeutic purposes. In fact, the line between these categories of use is not always clear [40–42], and many plants “used for food” also “serve to heal”. This idea is in tune with the broad concept of *health* as a state of complete physical, mental, and social well-being and not merely the absence of disease [43]. In urban agglomerations, shops called *dietéticas* (“health-food stores”) [44] are the focus of attention about the concept of *healthy food*, which was widespread by the media, and they are the places chosen to buy dietary supplements, functional foods, and nutraceuticals. 

The concept of functional foods is susceptible of different interpretations that referred to their characteristics, their active components, or their regulatory framework [45–48]. Overall, apart from their conventional value as a source of nutrients, functional foods provide benefits for certain body functions, important for the maintaining of health or to reduce disease risk [49, 50]. According to Kalra [51], functional foods are consumed for those purposes, but people are not aware of their specific components; however, they are recognized because they “are good for health”. The concept of *nutraceuticals* is also debatable. However, from the point of view of the consumer, nutraceuticals are functional foods that help to prevent a disease or collaborate in its treatment; therefore, their particular effects are recognized. In this context, it is noteworthy that what for a consumer is a functional food, for another one can act as a nutraceutical [51]. The categories presented in Table 1 derive from the consensus of informants.

Among immigrants, the integrative idea of “edible and healing plants” (functional foods and nutraceuticals included) is linked to their traditions, and it is invisible for the rest of the urban population. However, some functional foods and nutraceuticals prevalent within the immigrant community go on sale in the *dietéticas *(shops that are related to the nontraditional component of the UBK), and according to their diffusion level, spread by the media, they enter the general commercial circuit, and they become visible. The evaluation of this situation is an important methodological tool to understand the UBK dynamics: these health-food stores become *visualization *agents. 

### 2.3. Study Area and Involved Actors

 The Ciudad Autónoma de Buenos Aires or Capital Federal is placed over the West margin of the Río de la Plata in South latitude 34°36′ and West longitude 58°26′ [52]; it has an area of 202 km² and a population of 2,891,082 inhabitants [53]. Together with 24 departments of the Buenos Aires province, it forms the Great Buenos Aires [54, 55], with a total area of 3,833 km². These departments have a total population of 9,910,282 inhabitants [53]. In population terms, the Great Buenos Aires is the biggest conurbation of Argentina and the second in South America (after São Paulo metropolitan area and Mexico DF), the fifth of America, and the seventeenth in the world [56]. This large metropolitan area comprises strictly urban areas, some not urbanized areas with spontaneous vegetation, and transition areas between urban and rural sectors, named *periurban*, with mobile boundaries according to the urbanization rhythm and characterized by horticultural production that supplies the urban agglomeration [57–59].

The Bolivian immigration, caused especially for work reasons, settled first in the Northeast of the country, in the provinces of Jujuy, Salta, and Tucumán, working in the harvest. In the second half of the 20th century, their destinations diversified, and then, they settle again but this time in the metropolitan area of Buenos Aires, working with the horticulture in the periurban areas and the manufacturing industry, commerce, and the construction business in the urban areas [60, 61]. In 2001, 22% of the Bolivian population in Argentina (2,233,464 inhabitants according to that year census) was living in Jujuy and Salta, a low percentage compared to the 60% settled in Capital Federal (22%) and in the province of Buenos Aires (38%) that same year. 

The preference of recent immigrants to settle in metropolitan areas is also seen in the age structure of population: in Capital Federal and Buenos Aires province, the Bolivian immigrants over 54 years old are about 15% of the total population, and in Jujuy, they are over 43%. In Buenos Aires city, the immigrants coming from Bolivia, Peru, and Paraguay are the 5% of its total population, and in the whole country, they represent a little less than the 2% [62]. 

The called *horticultural belt* of Buenos Aires (Berazategui, Florencio Varela, and La Plata departments) supplies fresh vegetables to the inhabitants of the conurbation Buenos Aires-La Plata and other provinces. In 2001, 39.2% of the producers were Bolivian: of that total 75% are tenants, 25% owners that work almost exclusively with work force coming from their own country [63]. If it is considered that the first Bolivian immigrants, who arrived in the area about two or three decades ago [59, 63], worked as agricultural laborers, the social mobility of the group is marked [64].

### 2.4. Liniers Bolivian Market

The market of Bolivian immigrants that is the object of study of this research is placed in the neighborhood of Liniers, in Buenos Aires, and it is known as *Liniers market *(or *Bolivian market* for the population that does not belong to the segment). Liniers is one of the 48 neighborhoods or districts in which the Capital Federal is divided. It is located in the west of the city, its area is of 5.4 km², and its population is of 44,234 inhabitants. With regards to public urban and intercity transportation, the neighborhood is one of the main points of the city, with a lot of buses short- and long-distance routes that communicate the Capital Federal with departments of the Great Buenos Aires. Its central bus station is the second most important of the city, after Retiro. Likewise, there is a train station of Ferrocarril Sarmiento that links Buenos Aires city with the provinces of Buenos Aires, La Pampa, Córdoba, San Luis, and Mendoza, west to the country [52]. Surrounding this train station and Rivadavia Avenue (that goes through the city from East to West and it continues to the province of Buenos Aires), there is a commercial area with different shops, an important shopping mall, and the *Bolivian market.*

This market is a set of premises and street stalls that is specially concentrated in the street José León Suárez, one block away (a hundred meters) from the train station which is in the intersection of José León Suárez and Rivadavia Avenue. In the premises and street stalls, located in the sidewalk, food and medicinal vegetables and several products deriving from them are sold; there are also bars and restaurants of typical food and other shops of the sort in the cross and side streets. This receives the name of *market* or, sometimes, *fair*. In a broad sense, it is a market, because it is a site designed to sell products, either permanently or on specific days. However, it is different from other markets in the city, because the premises and stalls are not inside a building. On the other hand, it can be considered a fair, but local fairs are usually placed outdoors (streets and parks) and are held on specific days.

The activity is more intense in the Liniers market during the weekend (in fact the vehicular traffic is stopped), when a lot of people from all over the city and nearby cities of the province of Buenos Aires go there to purchase products as well as a tour and meeting place. The market is visited by the members of the Bolivian community that ask for specific products to preserve their own traditional recipes (dietary and therapeutic), members of the Peruvian immigrant community, for similar reasons, neighbors who are not part of these immigrants segments who find it a cheap place with a wide and diverse selection and purchase of food and therapeutic, and, finally, some people from other neighborhoods of the city and different social sectors that have started to use this market as a place to buy functional foods and nutraceuticals.

### 2.5. Methodological Design

 Generally, for researches on traditional markets, the methodology proposed by Cunningham [3] has been followed. 30 outlets (street stalls and premises) have been surveyed, where samples of different vegetal elements were gathered. They have been placed, to document the work, in the collections of LEBA and the herbarium samples in the Herbario LP, División Plantas Vasculares, Museo de La Plata. For the species nomenclature, database of different institutions were followed for reference purposes [65, 66]. Ethnobotanical data were obtained according to usual qualitative methods [67–70], especially, using simultaneously participant observation (to record the plants actually marketed) and interviews (both open and semistructured ones), besides the literature review relevant to the observed plant elements. Questions were designed to obtain information concerning the name of species at the market, the parts of the plant commercialized, and the use(s) attributed to each of them. These procedures always performed with the consent of the informants. Data was registered following the parameters used in other studies about urban markets [7, 8, 71]. For some products, the information from labels and inserts was also assessed that for the general public, these directs the selection of products to consume.

Informants were interviewed on the basis of saturation of information, so 50 market sellers (from a total of 95 salesmen) of both sexes and different ages have been included. They are considered *qualified informants*: immigrants that expressed their knowledge about the characteristics and properties of different vegetal elements and the way they are used. They showed a positive attitude to provide the requested information. 

The record of the gathered data for plants that are sold in the Bolivian market of Liniers pointed to three distinct categories.

1. *Exclusive *items of the Bolivian immigrant segment are not found in the general commercial circuit, and they satisfy the characteristic needs of this group, which allows identifying the UBK linked to traditions.
2. *Generalized* items which are also found in the general circuit (supermarkets, greengrocers, herbalist's shops, and *dietéticas*), and consequently, they are related to the nontraditional UBK.
3. *Frequent* items in the immigrants setting that are also found in the general circuit but their presence is sporadic or, at least, they do not are very well known. However, they are sold in *dietéticas*, so these items are related to the UBK dynamics. The frequency within the market refers to the fact they are for sale in all the analyzed premises and street stalls.

For the purpose of this research, the generalized items were not considered, because they are visible for everyone. The focus is placed on the exclusive items (invisible) and the frequent ones (in process of visualization).

## 3. Results and Discussion

Until the moment, 160 edible species commercialized in Liniers Bolivian market have been surveyed (vegetables, legumes, fresh and dry fruit, condiments, and beverage flavorings). Of that total, products or part of plants considered functional foods or nutraceuticals, exclusive or frequent within the market, belonging to 54 species of 19 botanical families are sold. These are shown in Table 1, organized by families, in alphabetical order. The table also includes the local name, parts of the plant or products obtained from them, their uses, product situation: exclusive or frequent (under the terms defined above), and samples obtained and deposited in LEBA and LP under the leg. J. A. Hurrell et al. (JH) and F. Buet Costantino et al. (FB). 

It is relevant to remember that the exclusive or frequent character concerns to the products and not to the species. In this way, the “arrope de higo” (“fig syrup”), *Ficus carica* L., is exclusive of the market but the edible fresh or dry fruits or the jams, which are also available in Liniers, are products with a wide diffusion in the general commercial circuit, so they are *generalized*, according to the categories of use. In the case of *Phaseolus vulgaris* L., the common bean and the ones called “alubia”, “colorado” (red), “negro” (black) and “regina”, among others [35], are frequent in the market (and consequently, they are available in the general circuit but generally they are sold packed); meanwhile, another beans called “canario” and “panamito” are exclusive of the Liniers market, where they are only sold loose.

### 3.1. Origin of Functional Foods and Nutraceuticals

The fresh vegetables commercialized in Liniers market are cultivated in homegardens of the conurbation periurban areas; most of them are situated in the Buenos Aires horticultural belt. This information was given by the interviewed informants, but it was also taken from researches about peri-urban homegardens developed in other research lines of the LEBA [63, 72–74]. In some cases, like *Porophyllum ruderale *(Jacq.) Cass., “quirquiña”, and *Tagetes minuta *L., “huacatay”, they were cultivated with seeds brought from Bolivia. Dry or manufactured products usually come from Bolivia as well, even if the origin of the product is different; for example, the leaves of *Cynara cardunculus *L., “alcachofa”, of the tea bags and the pills that are sold in street stalls of Liniers are products originated in Peru but they have entered from Bolivia. 

All the informats agree that the vegetal elements come from Bolivia and not from the Argentinean Northeast even if this region belongs to the same ecological and cultural Andean unit and the same edible and therapeutic plants are consumed; this is the case of, for example, the Andean edible roots *Smallanthus sonchifolius *(Poepp. and Endl.) H. Rob., “yacón”, and *Pachyrhizus ahipa *(Wedd.) Parodi, “ajipa” [75–80].

### 3.2. Contexts of Circulation of Functional Foods and Nutraceutics

There are some fresh aerial parts of plants that are exclusive from Liniers market, like *Baccharis articulata *(Lam) Pers. “carqueja”; *B. trimera* (Less.) DC., “carquejilla”; *Matricaria recutita *L., “manzanilla”; *Borago officinalis *L., “borraja”, *Dysphania ambrosioides *(L.) Mosyakin and Clemants, “paico”, *Melissa officinalis *L., “toronjil”; *Mentha *x* piperita *L., “menta”, *M. spicata* L., “yerba buena”, *Rosmarinus officinalis *L., “romero”, *Eucalyptus cinerea *F. Muell. *ex* Benth., “eucalipto”, *Aloysia citriodora *Palau, “cedrón” and *A. polystachya *(Griseb.) Moldenke, “burrito”. Nevertheless, they are also part of within-family product exchange, and they are also available from direct harvest in the conurbation periurban and nonurban areas. Of the species listed, the dry aerial parts are sold as an herbalist product in the general commercial circuit. In contrast, the aerial parts of *Porophyllum ruderale *and *Tagetes minuta *are not available in herbalist's shops and *dietéticas*. The fresh aerial parts of *Coriandrum sativum *L. are only commercialized in Liniers market (they are rarely found in the general circuit), and they are named “cilantro”; on the other hand, their mericarps, called “semillas” are named “coriandro”, and they are widespread in supermarkets, *dietéticas *and spices' shops. In Liniers market, the whole plant of* Stevia rebaudiana *L., “yerba dulce”, is sold, while in the *dietéticas, *there are only available packed products: in bags, liquid, or powder.

Fresh products which are exclusive of this Bolivian market are the edible fruits of the Cucurbitaceae: *Cyclanthera pedata *(L.) Schrader, “caiwa” or “achojcha”, *Sechium edule *(Jacq.) Sw., “chayote” or “papa del aire”, and the *Cucurbita ficifolia *Bouché, “cayote”, or “alcayote”. The last example is sporadically available in greengrocer, and the jam made from its pulp is sometimes sold in supermarkets, because it is from time to time cultivated in the homegardens of the periurban area [35, 36, 72, 78]. Among the Solanaceae, there are some varieties of *Capsicum annuum* L. that are exclusive of Liners market like (various fruits of different color that are offered fresh or dry, not powdered); and *C. pubescens* Ruiz and Pav., “locoto” or “rocoto” (fresh fruit and packed powder product). Other varieties of *C. annuum, *like peppers, fresh chilies, and condiment in powder (ground chili and paprika), are generalized vegetal elements. Of *Solanum tuberosum* L., “papa”, are exclusive of Liners market the dry tubers called *chuño*, from both “blancas” (white) and “negras” (black) potatoes; while the fresh potatoes are available in the general commercial circuit [35], other small tubers of this species called “papines”, are a frequent item in Liniers, but they are not exclusive: sometimes they are found in some greengrocer around the city.

Andean microthermal tubers, *Ullucus tuberosus *Caldas, “papa lisa” or “ulluco”, and *Oxalis tuberosa* Molina, “oca”, are valued nowadays due to their antioxidant properties [81], they are sold fresh, and they are frequent in Liniers market; sporadically, they can also be seen in some greengrocer of the general circuit. Occasionally, in the past, tubers of *Tropaeolum tuberosum* Ruiz and Pav., “isañu” or “añu” were sold in Liniers market, but they stopped selling them because there were no buyers; according to an informant, they have anaphrodisiac effects (apparently were used for this purpose by the soldiers of the Inca Empire), so people dismiss them [82, 83].

Among the Poaceae, *Cymbopogon citratus *(DC.) Stapf, “pasto limón” (“lemon grass”), the fresh tillers are sold in Liniers market and sporadically in the general circuit. Different varieties of “maíz”, *Zea mays* L., are exclusive of the Bolivian market, like the “maíz morado” (“purple corn”), that are sold dry, whole, or in powder to make “chicha morada” (a refreshing beverage of Peru). The dry styles, called “barba de choclo”, is frequent in the market and also in some herbalist's shops. Fresh corn is a generalized item.

Arrope (syrup) from fruits of *Ficus carica*, *Opuntia ficus-indica* L. and *Vitis vinífera* L. are exclusive products of Liniers market; however, the fresh and dry fruits of these species are available both in Liniers and in the general circuit. On the other hand, arrope of “algarrobo”, *Prosopis alba* Griseb., and of “chañar”, *Geoffroea decorticans *(Gillies *ex* Hook. and Arn.) Burkart are also sold in *dietéticas*. A flour from the fruits of *Prosopis alba* is available in Liniers and in *dietéticas*. The bark of “chañar” is sold as a therapeutic product in herbalist's shops, with similar effects as its syrup [2, 36]. The husk of the seed *Coffea arabica *L., “café”, called “sultana” is only available in Liniers.

*Lepidium meyenii* Walp., “maca”, *Plukenetia volubilis* L., “sacha inchi”, and *Morinda citrifolia* L., “noni”, in different products but mostly in powder are very frequent in Liniers, and lately, they are also available in *dietéticas*, quickly spreading in the general commercial circuit. “Maca” and “sacha inchi” come from Peru, where they have been known since Pre-Inca times [84, 85]. On the contrary, “noni” comes from Polynesia, from there it entered Peru, then it went to Bolivia and then to Argentina. It has had world diffusion because of the wide range of therapeutic effects are attributed to it [86].

The legumes that are sold in Liniers usually are found in the general circuit, but the dry seeds and flours obtained from some of them are less frequent; in Liniers market, they are frequent and they are sold loose. This is the case of the *Cicer arietinum* L., “garbanzo”; *Glycine max *(L) Merr., “soja”; *Lens culinaris *Medik., “lenteja”, and its varieties, “lentejón”, “lenteja turca” and “lenteja canadiense”; *Lupinus albus* L., “lupín”; *Phaseolus lunatus* L., “poroto de manteca” and “pallar”; *Pisum sativum *L., “arveja”; *Vicia faba* L., “haba”; *Vigna angularis *(Willd.) Ohwi and H. Ohashi, “poroto adzuki”; *Vigna unguiculata *(L.) Walp., “poroto tape” o “caupí” and *Vigna radiata *(L.) R. Wilczer, “poroto mung”. The sprout of this last species is available in Buenos Aires as “brotes de soja” which are spread widely [35]. Enclusive of Liniers is the fresh legumes of *Lablab purpureus* (L.) Sweet, “poroto japonés”, and the dry seeds of *Lupinus mutabilis* Sweet, “tauri” o “tarwi”; this one is an important Andean crop due to its high protein value [80, 83, 87] and the seeds of a variety of *Arachis hypogaea *L. called “maní boliviano” (“Bolivian peanut”) [88]. The case of *Phaseolus vulgaris* has been mentioned before. *Glycyrrhiza glabra *L., “regaliz”, is sold as therapeutic and as sweetener in Liniers and in some *dietéticas* and herbalist's shops.

The preserved pulp of *Tamarindus indica *L., “tamarindo”, is available in Liniers market and, occasionally, in the general commercial circuit. It is interesting to notice that this same product is also commercialized in shops owned by Chinese immigrants, which are concentrated in another neighborhood of Buenos Aires, Belgrano, and the set of shops as a whole is called “barrio chino” (“Chinese neighborhood”). The same happens with the dry sees (sold loose) of the species of *Vigna*: “poroto adzuki”, “poroto mung” and “poroto tape” [34–36]. The presence of these in two immigrant segments so different in origin and traditions within the same conurbation opens a future comparison, taking into account the UBK component linked to traditions.

Even though it has not been dealt in this paper, a project is being developed, within LEBA, which aims to the understanding of the setting of exchange networks. The informants describe clearly defined routes to obtain the products that enable obtaining them very quickly (48–72 hours from their origin place, in Bolivia, to their outlet place in the Liniers market), and they make the selling of perishable products feasible. However, it has to be highlighted the incorporation of the plants that provide these fresh products so they can be incorporated to the stock-in-trade of the periurban vegetable gardens, which results in an increase in the agrobiodiversity of the area.

## 4. Conclusions

The research developed in Liniers market, identified with the segment of Bolivian immigrants (a group with a long history in Argentina but with a recent presence in the metropolitan area of Buenos Aires), enables a characterization of the component of the UBK linked to traditions and its own dynamics. Besides, this market being placed in an urban area suggests that traditional markets are an important source of plant diversity, which adds variety and choices to the UBK, process that takes place in a short time and that is spread quickly to the rest of the population. In this way, elements previously invisible become visible. Considering *dietéticas *as visualization agents is a relevant methodological contribution for Urban Ethnobotany, because it helps to understand the BK dynamics in large metropolitan areas.

On one hand, typical elements of a community are incorporated through the market; in this particular case, well-known Andean crops, like “papines” (*Solanum tuberosum*), “oca” (*Oxalis tuberosa*), “ulluco” (*Ullucus tuberosus*), “yacón” (S*mallanthus sonchifolius*), “ajipa” (*Pachyrhizus ahipa*), “tauri” (*Lupinus mutabilis*), “caiwa”, (*Cyclanthera pedata*), and “cayote” (*Cucurbita ficifolia*), and, likewise, the market imply a quick entrance of several exotic elements (both to the city and to the immigrant group), which becomes easier due to the informality of the products circulation; for example: the “noni” (*Morinda citrifolia*). Besides, sometimes several fresh product are incorporated to local horticulture to enhance their availability, thus promoting agrobiodiversity. On the other hand, a special mention about the use of vegetables and products deriving from them which are available in the Liniers market in terms of functional foods and nutraceuticals. The usage of food with therapeutic ends is not new, it has been part of human knowledge since ancient times, and it is present in several cultures, that is why it has been an object of study to professional of different areas of knowledge, including, especially in recent years, ethnobotany [40–42, 89, 90]. In the last three decades, this concept about “edible and healing plants” has been globalized and a renewed interest can be seen about the healing properties of food and the products that are known as *dietary supplements*, which are added to different substances so they are beneficial to the health. In this sense, traditional markets are relevant places to acquire functional foods and nutraceuticals, this is why they can satisfy the needs of regular consumers (members of the community) and, at the same time, respond to the demands of the pluricultural conglomerate in which they are immersed.

## Figures and Tables

**Table 1:** Exclusive and frequent functional food and nutraceuticals in the market of Bolivian immigrants in Liniers, Ciudad de Buenos Aires, Argentina.

Families/species	Local name	Parts/products	Uses	Situation	Samples
Apiaceae					
*Coriandrum sativum *L.	Cilantro	Fresh leaves	Food and condiment, as a substitute of parsley. Nutraceutical: diuretic, aperitive, digestive, and antispasmodic	Frequent	FB 430 (LEBA)

					
Asteraceae					
*Baccharis articulata * (Lam) Pers.	Carqueja	Fresh aerial parts (in bunches)	Beverage flavouring. Nutraceutical: tonic, digestive, hepatic, diuretic, febrifuge, and cordial; in external application, vulnerary	Exclusive	FB 416 (LP)
*Baccharis trimera *(Less.) DC.	Carquejilla/ Carqueja	Fresh aerial parts (in bunches)	Same as above	Exclusive	FB 424 (LEBA)
*Cynara cardunculus *L. (= *C. scolymus *L.)	Alcachofa	Leaves in bags and pills	For infusions. Nutraceutical: hepatic, cholagogue, choleretic, and depurative	Exclusive	JH H093, H094 (LEBA)
*Matricaria recutita *L.	Manzanilla	Fresh aerial parts (in bunches)	Beverage flavouring. Nutraceutical: sedative, slimming, digestive, antispasmodic, emmenagogue, pectoral, emollient, and vermifuge	Exclusive	FB 427 (LP)
*Porophyllum ruderale *(Jacq.) Cass.	Quirquiña	Fresh aerial parts (in bunches)	Food and condiment, for soups, stews and sauces. Nutraceutical: diaphoretic, antispasmodic; in external application, vulnerary	Exclusive	FB 413 (LP)
*Smallanthus sonchifolius* (Poepp. & Endl.) H.Rob.	Yacón	Fresh roots and jams	Food, as fruit or in salads (raw), for juices, syrups, jams, and teas. Functional food or Nutraceutical: antidiabetic	Exclusive	JH 6891 (LP), L006 (LEBA)
*Stevia rebaudiana *L.	Yerba dulce	Fresh aerial parts or whole plant	Sweetener, for infusions and confectionary. Nutraceutical: antidiabetic and “antiageing” (antioxidant)	Exclusive	FB 415 (LP)
*Tagetes minuta *L.	Huacatay	Fresh aerial parts (in bunches)	Condiment for soups, stews and sauces. Nutraceutical: diuretic, digestive, and antispasmodic. Insecticide	Exclusive	FB 403 (LEBA)

					
Basellaceae					
*Ullucus tuberosus *Caldas	Papa lisa/ Ulluco	Fresh tubers (sold loose or packed)	Food, for soups, stews, locro, and purees. Functional food: “healthy” food (antioxidant)	Frequent	FB 439 (LEBA)

					
Boraginaceae					
*Borago officinalis *L.	Borraja	Fresh aerial parts	Food, eaten as a vegetable or in patty fillings; condiment in sauces, soups, and stews. Functional food or nutraceutical: expectorant, cordial	Frequent	FB 414 (LP)

					
Brassicaceae					
*Lepidium meyenii* Walp. (=* L peruvianum *G. Chacón)	Maca	Roots in powder or as flour (sold loose or packed)	Nutraceutical: tonic of the nervous system, to stimulate memory, to improve sexuality and fertility, against fatigue and stress, “antiageing” (antioxidant). It is added to food and drinks	Frequent	JH H091, H160 (LEBA)
					
Cactaceae					
*Opuntia ficus-indica * (L.) Mill.	Tuna	Arrope (syrup) (in bottles)	Food. Functional food o nutraceutical: diuretic, antispasmodic, emollient, and vermifuge	Exclusive	JH L002 (LEBA)

					
Chenopodiaceae					
*Dysphania ambrosioides *(L.) Mosyakin & Clemants (= *Chenopodium ambrosioides* L.)	Paico	Fresh aerial parts (in bunches)	Condiment in soups, stews, and other foods. Beverage flavouring. Nutraceutical: tonic, aperitive, febrifuge, digestive, antispasmodic, carminative, hypotensive, emmenagogue, and vermifuge; in external application, antihemorroidal	Exclusive	FB 419 (LP)

					
Cucurbitaceae					
*Cyclanthera pedata *(L.) Schrader	Caiwa/ Achojcha	Fresh fruits.	Food, used as pumpkin, in stews and soups. Functional food or nutraceutical: antidiabetic, analgesic, and hypotensive	Exclusive	FB 417 (LEBA)
*Cucurbita ficifolia *Bouché	Cayote/ Alcayote	Fresh fruits	Food, as a fruit; also in soups and stews. Functional food or nutraceutical: antidiabetic	Exclusive	JH 565 (LP)
*Sechium edule * (Jacq.) Sw.	Chayote/ Papa del aire	Fresh fruits	Food, in stews, soups, fried, pies, and jams. Functional food or nutraceutical: diuretic, antidiabetic, and hypotensive	Exclusive	FB 418 (LEBA)

					
Euphorbiaceae					
*Plukenetia volubilis* L.	Sacha inchi	Seeds in snacks, liquid, ointment and in powder	Food. Nutraceutical: depurative, hypocholesterolemic, antioxidant; in ointment, for bone pain, and inflammation	Frequent	JH L029 (LEBA)

Lamiaceae					
*Melissa officinalis *L.	Toronjil/ Melisa	Fresh aerial parts	Condiment for sauces and various dishes. Nutraceutical: digestive, carminative, antispasmodic, cordial, and emmenagogue	Exclusive	FB 422 (LEBA)
*Mentha *x* piperita *L.	Menta	Fresh aerial parts	Condiment for various dishes. Beverage flavouring. Nutraceutical: aperitive, stimulant, digestive, antidiarrheal, carminative, and vermifuge	Exclusive	FB 428 (LEBA)
*Mentha spicata* L.	Yerba buena	Fresh aerial parts	Condiment for various dishes. Beverage flavouring. Nutraceutical: stimulant, digestive, hepatic, cholagogue, and pectoral	Exclusive	FB 423 (LEBA)
*Rosmarinus officinalis *L.	Romero	Fresh aerial parts	Condiment for various dishes. Nutraceutical: antispasmodic, digestive, hepatic, depurative, and emmenagogue	Exclusive	FB 457 (LEBA)

					
Leguminosae					
*Arachis hypogaea *L.	Maní boliviano	Dry seeds (sold loose)	Food, for soups and stews. Functional food or nutraceutical: laxative, emollient, and pectoral	Exclusive	JH L007 (LEBA)
*Cicer arietinum *L.	Garbanzo	Dry seeds and flour (sold loose)	Food, for soups, stews, and side dishes. Functional food or nutraceutical: diuretic, hypocholesterolemic	Frequent	JH L027 (LEBA)
*Geoffroea decorticans *(Gillies *ex* Hook. & Arn.) Burkart	Chañar	Arrope (syrup) (in bottles)	Food. Nutraceutical: antitussive, expectorant, anticatarrhal, balsamic, emollient, and antiasthmatic	Frequent	JH L005 (LEBA)
*Glycyrrhiza glabra *L.	Regaliz	Dry chopped roots	Sweetener. Nutraceutical: anti-inflammatory, digestive, antispasmodic, hepatic, diuretic, emollient, laxative, expectorant, and antiasthmatic	Frequent	JH H084 (LEBA)
*Glycine max *(L) Merr.	Soja	Dry seeds and flour (sold loose)	Food, for stews, soups, and salads. Functional food or nutraceutical: diuretic, hypocholesterolemic, digestive, and laxative	Frequent	JH L019 (LEBA)
*Lablab purpureus * (L.) Sweet	Poroto japonés	Fresh beans	Food (cooked). Functional food or nutraceutical: astringent, antidiarrheal, digestive, and febrifuge	Exclusive	FB 404 (LEBA)
*Lens culinaris *Medik.	Lenteja común	Dry seeds (sold loose)	Food, for soups and stews. Functional food or nutraceutical: antianemic, digestive, and laxative	Frequent	JH L018 (LEBA)
	Lentejón		Same as above	Frequent	JH L017 (LEBA)
	Lenteja *turca *		Same as above	Frequent	FB H05 (LEBA)
	Lenteja *canadiense *		Same as above	Frequent	JH L013 (LEBA)
*Lupinus albus* L.	Lupín	Dry seeds (sold loose)	Food, for soups and stews. Functional food or nutraceutical: diuretic, vermifuge, emmenagogue	Frequent	JH L014 (LEBA)
*Lupinus mutabilis* Sweet	Tauri/Tarwi	Dry seeds (sold loose)	Food, for soups, stews, purees, tamales, humita, and tortillas. Functional food or nutraceutical: diuretic, emollient, and vermifuge	Exclusive	FB H14 (LEBA)
*Pachyrhizus ahipa * (Wedd.) Parodi	Ajipa	Fresh roots	Food, as fruit (raw) or vegetable (cooked). Functional food or nutraceutical: diuretic, expectorant, and antitussive	Exclusive	FB 374 (LEBA)
*Phaseolus lunatus* L.	Poroto pallar	Dry seeds (sold loose)	Food, for salads, soups, and stews. Functional food or nutraceutical: astringent, febrifuge, and emollient	Frequent	FB H13 (LEBA)
	Poroto de manteca		Same as above	Frequent	JH L024 (LEBA)
*Phaseolus vulgaris* L.	Poroto/ Chaucha	Dry seeds and fresh legumes (sold loose)	Food, for salads, soups, stews, and locro. Functional food or nutraceutical: diuretic, hypoglycemic, hypotensive, and resolutive	Frequent	JH L028 (LEBA)
	Poroto *alubia *		Same as above	Frequent	JH L023 (LEBA)
	Poroto *negro *		Same as above	Frequent	JH L021 (LEBA)
	Poroto *colorado *		Same as above	Frequent	JH L022 (LEBA)
	Poroto *regina *		Same as above	Frequent	JH L008 (LEBA)
	Poroto *cranberry *		Same as above	Frequent	JH L010 (LEBA)
	Poroto *San Francisco *		Same as above	Frequent	FB H11 (LEBA)
	Poroto *pitai *		Same as above	Frequent	FB H12 (LEBA)
	Poroto *paraguayo *		Same as above	Frequent	JH L020 (LEBA)
	Poroto *canario *		Same as above	Frequent	FB H10 (LEBA)
	Poroto* panamito *		Same as above	Exclusive	FB 443 (LEBA)
*Pisum sativum *L.	Arveja	Dry seeds and flour (sold loose)	Food, for salads, soups, and stews. Functional food or nutraceutical: digestive, febrifuge, against dermatosis, and contraceptive	Frequent	JH L026 (LEBA)
*Prosopis alba *Griseb.	Algarrobo blanco	Arrope (syrup) (in bottles) and flour (sold loose).	Food. Nutraceutical: stomachic, laxative, diuretic, pectoral, and antiasthmatic	Frequent	JH L004 (LEBA)
*Tamarindus indica *L.	Tamarindo	Fruit pulp.	Food and Condiment. Functional food or nutraceutical: digestive, refreshing, laxative, and purgative	Frequent	JH L001 (LEBA)
*Vicia faba *L.	Haba	Dry and toasted seeds (snacks) and fresh legumes (sold loose)	Food, for salads, soups and stews. Functional food or nutraceutical: diuretic, emollient, resolutive, and against colds	Frequent	JH L015, L016 (LEBA)
*Vigna angularis * (Willd.) Ohwi & H. Ohashi	Poroto adzuki	Dry seeds (sold loose)	Food, for soups and stews, with cereals and rice, and confectionary. Functional food or nutraceutical: digestive, laxative, and hypoglycemic	Frequent	JH H101 (LEBA)
*Vigna radiata * (L.) R. Wilczer	Poroto mung	Dry seeds (sold loose)	Food, for soups and stews. Functional food or nutraceutical: digestive, antidiarrheal, febrifuge, and tonic.	Frequent	JH L011 (LEBA)
*Vigna unguiculata *(L.) Walp.	Poroto tape/Caupí	Dry seeds (sold loose)	Food, for soups, stews and purees. Functional food or nutraceutical: diuretic, digestive, laxative, tonic, and galactogene	Frequent	JH L012 (LEBA)

					
Moraceae					
*Ficus carica *L.	Higo	Arrope (syrup) (in bottles)	Food. Functional food or nutraceutical: anti-inflammatory, emollient, vermifuge, and antioxidant	Exclusive	FB 453 (LEBA)

					
Myrtaceae					
*Eucalyptus cinérea *F. Muell. *ex* Benth.	Eucalipto	Branches with fresh leaves (in bunches)	Beverage flavouring. Nutraceutical: expectorant, against colds, anticatarrhal, antitussive, antiasthmatic, and antirheumatic	Exclusive	FB 425 (LEBA)

					
Oxalidaceae					
*Oxalis tuberosa* Molina	Oca	Fresh tubers (sold loose or packed)	Food, for soups, stews, purees (cooked). Functional food: “healthy” food (antioxidant)	Frequent	FB 438 (LEBA)

					
Poaceae					
*Cymbopogon citratus *(DC.) Stapf	Pasto limón/ Citronela	Fresh tillers (in bunches)	Condiment for food and beverage flavouring. Nutraceutical: sedative, stomachic, carminative, and antidiarrheal	Frequent	FB 407 (LEBA)
*Zea mays* L.	Maíz *morado* (*kulli*)	Whole dry spikes or in powder (sold loose)	To make *chicha morada *(refreshing drink). Nutraceutical: depurative, hypotensive, anti-inflammatory, and antioxidant	Exclusive	FB 431 (LEBA)
	Maíz *chuspillo *	Dry grains (sold loose or packed)	For toasted corn. Functional food	Exclusive	FB 448 (LEBA)
	Maíz *huillcaparu *		To make *chicha* (alcoholic beverage). Functional food	Exclusive	FB 447 (LEBA)
	Maíz *colorado *		For soups, stews and other dishes. Functional food	Exclusive	FB 450 (LEBA)
	Maíz *blanco *		Same as above	Exclusive	FB 449 (LEBA)
	Maíz *mote *o* pelado *	Dry or cooked grains (sold packed)	For stews and other dishes, boiled in water. Functional food	Exclusive	FB 451 (LEBA)
	Barba de choclo	Dry styles (sold loose)	Diuretic, hepatic, and antinephritic.	Frequent	JH H163 (LEBA)

Rubiaceae					
*Coffea arabica *L.	Sultana	Seeds (seed coat) (sold loose or packed)	Beverage flavouring. Nutraceutical: antidiabetic, stimulant, antinephritic, and febrifuge	Exclusive	JH C095 (LEBA)
*Morinda citrifolia* L.	Noni	Pulp made flour or powder (loose or packed) and capsules	To make beverages or to add to infusions. Nutraceutical: stimulant of immune system, antidepressant, sleep regulator, “antiageing” (antioxidant), anti-inflammatory, and cordial	Frequent	JH H092, H161-H162 (LEBA)

					
Solanaceae					
*Capsicum annuum *L.	Ají *picante *	Fresh and dry fruits (sold loose or packed)	Food and condiment, for sauces, soups, stews, side dishes, and patty fillings. Functional food or nutraceutical: tonic, analgesic, and stimulant of the digestive system	Exclusive	JH C096 (LEBA)
	Ají *escabeche *			Exclusive	FB 433 (LEBA)
	Ají* amarillo *			Exclusive	FB 435 (LEBA)
	Ají *campanita *			Exclusive	FB 421 (LEBA)
*Capsicum pubescens* Ruiz & Pav.	Locoto/ Rocoto	Fresh fruits (sold loose) and in powder (packed)	Condiment for soups, sauces, stews, patty fillings, and various dishes. Functional food or nutraceutical: tonic, analgesic, and stimulant of the digestive system	Exclusive	JH C094 (LEBA)
*Solanum tuberosum* L.	Papa blanca, papa negra	Dried tubers (*chuño*) (sold loose or packed)	Food, for various dishes. Functional food and nutraceutical: cordial, hypotensive, and antispasmodic	Exclusive	FB 441-442 (LEBA)
	Papines	Fresh tubers (sold loose or packed)	Same as above	Frequent	FB 440 (LEBA)

Verbenaceae					
*Aloysia citriodora *Palau	Cedrón	Fresh aerial parts or whole plant	Food, condiment and beverage flavouring. Nutraceutical: against gastrointestinal disorders, sedative, and hypotensive	Exclusive	FB 455 (LEBA)
*Aloysia polystachya *(Griseb.) Moldenke	Burrito/ Burro	Fresh aerial parts or whole plant	Beverage flavouring, fresh leaves are added to mate or used for make medicinal teas. Nutraceutical: sedative, digestive, antispasmodic, hepatic, carminative, and antiemetic	Exclusive	FB 456 (LEBA)

Vitaceae					
*Vitis vinífera* L.	Uva	Arrope (syrup) (in bottles)	Food. Nutraceutical: refreshing, diuretic, astringent, and antidiarrheal	Exclusive	FB 454 (LEBA)
